# Beyond group engagement: Multiple pathways from encounters with the police to cooperation and compliance in Northern Ireland

**DOI:** 10.1371/journal.pone.0184436

**Published:** 2017-09-07

**Authors:** Samuel Pehrson, Lee Devaney, Dominic Bryan, Danielle L. Blaylock

**Affiliations:** 1 School of Psychology and Neuroscience, University of St Andrews, St Andrews, United Kingdom; 2 School of Psychology, Queen’s University Belfast, Belfast, United Kingdom; 3 Institute of Irish Studies, Queen’s University Belfast, Belfast, United Kingdom; 4 School of Social Sciences, Education and Social Work, Queen’s University Belfast, Belfast, United Kingdom; Fordham University, UNITED STATES

## Abstract

In a sample of young people in Northern Ireland (N = 819), we examine the relationships between the quality of experience with police officers and police legitimacy. We examine potential pathways through which experiences may either support or undermine the legitimacy of the police, and thus cooperation and compliance with them. We find evidence that perceptions of the police as having goals that align with those of wider society, and as being fair in general, mediate relations between the quality of encounters and legitimacy, which in turn mediates the relation with cooperation and compliance. Identification with wider society was not a reliable mediator, contrary to our predictions based on the Group Engagement Model. Moreover, our analysis of the structure of police fairness perceptions finds no support for the distinction between procedural and distributive police fairness as usually conceived. Implications for the social psychological understanding of legitimate authority are discussed.

## Introduction

Legitimate authority is a form of social power that involves voluntary submission [1–3]. Being voluntary, there is no need for coercive threats or rewards to induce deference to a legitimate authority [3,4]. At the same time, being a kind of submission, legitimate authority does not require one to be convinced of the appropriateness of a specific course of action, distinguishing legitimacy from persuasion. It is clear that legitimacy lies at the heart of many facets of human social relations, including cooperation and compliance with the law and law enforcement agencies [5,6].

Theorists tend to agree that legitimacy takes the form of a normative sense of obligation to obey a given authority, thus either explicitly or implicitly placing its operation within psychological group processes [3,4,7]. However, merely to describe legitimacy as a group norm of obedience to a given authority is insufficient as an explanation: Why is it normative to obey some actors and roles but not others? How is legitimacy gained and lost? Such questions are typically approached in terms of fairness, both in terms of a general perception of particular authorities as being fair or not, and in terms of direct experiences of fair or unfair treatment [1]. As we explain below, there is considerable evidence that people draw on their experiences of particular authorities when assessing their legitimacy, and that fairness is central [1,6,8–12].

Within existing theoretical frameworks this is understood to be because being unfairly treated by an authority undermines one’s identity as a member of the group associated with that authority, leading to a withdrawal of cooperation with them–what might be termed a dis-identification pathway [7,13]. We aim to extend this account by considering this process along with two alternative pathways. First, people may exclude the authority from the group, thus undermining legitimacy, rather than dis-identifying themselves. Second, incumbents such as police officers may violate the normatively accepted constraints on which their designation as legitimate wielders of authority is conditional. We elaborate on these mechanisms below before examining them empirically in context of police-youth relationships in Northern Ireland.

### Fairness as the primary antecedent of police legitimacy

Empirical evidence supporting the key role of authority fairness in promoting legitimacy is broad and extensive. Notable studies on interactions with the police and police legitimacy include major surveys of citizens in New York [6,11,12,14], Chicago [15], Oakland and Los Angeles [10,16] and London [8,9]. All support the view that experiences of fair police conduct improve perceptions of the police, as does similar survey work and conducted elsewhere [17,18]. Further evidence from a national survey conducted in the United States shows that the fairness of police conduct matters more to people than the favourability of outcomes following traffic stops [19], while analysis of archival crime data from New York City finds variations in violent crime over time to be systematically related to unfair police conduct [20]. The fairness judgements affecting legitimacy need not necessarily be directed towards specific concrete encounters with the police but can also take the form of more abstract judgements of police fairness [6,11,12,14,16,17,21]. Fairness also underpins the perceived legitimacy of courts [10,16], prison authorities [22] and governments [2].

Scholars frequently distinguish between distributive fairness, which entails equality of treatment and outcomes, and procedural fairness, which includes various aspects of police decision making such as impartiality and consideration of the views of those they are dealing with [5,15]. Respectful interpersonal treatment is often added to this as a further aspect of procedural fairness [6,14,23]. The effects of procedural and distributive justice are argued to be distinct, with procedural justice determining the quality of the relationship with authorities, and distributive fairness operating instead via an instrumental pathway [5,11]. Multiple regression models pitting procedural against distributive fairness in predicting outcomes such as legitimacy and cooperation frequently find both to be reliable predictors, but procedural fairness to be the stronger and more consistent of the two [11,15,16]. On the basis of such findings, it is argued that it is the fairness of processes rather the equality of outcomes for different groups that underpins the legitimacy of authorities such as the police.

However, the manner in which procedural versus distributive fairness are usually measured has been subject to criticism that casts some doubt on the validity of the sharp theoretical distinction that is often drawn between them. For example, Reisig [23] note that measurement properties of the scales are usually evaluated using only the Cronbach alpha coefficient, which is not an appropriate tool for assessing the dimensionality of constructs. They find that factor loadings of items commonly used to measure the procedural and distributive fairness of the police–for example items used by Sunshine and Tyler [11,12], Tyler [15] and Tyler and Huo [16]—do not support the theorised distinction, with several loadings of procedural items on the ‘distributive’ factor and vice versa, and a correlation of .69 between the two. Similarly, a meta-analytic investigation of the relationship between procedural and distributive fairness finds an average correlation of .64 [24]. This value varies substantially between studies, however, and is likely to be underestimated due to measurement error. In line with this, a correlation of .89 between procedural and distributive police fairness has been reported when modelling them as latent factors in order to account for unsystematic measurement error [25]. Hauenstein et al. [24] strongly advise researchers to measure general fairness rather than use such closely related sub-dimensions in ‘predictive’ research (i.e. studies in which fairness is used to predict outcomes in regression models). Strong correlations between predictors in a regression model have the statistical consequence of forcing their respective regression weights apart from each other, which risks overstating the importance of one over the other.

The procedural-distributive distinction can also be criticised in more experiential terms, with some arguing that it is general fairness rather than specific types of fairness that is meaningful to participants, and that this is what drives behaviour [26,27]. The distinction, then, may be “more semantic than real” [24]. To the extent that these two kinds of fairness are closely related in practice, it is quite possible that the relative devaluing of distributive fairness in the literature is unwarranted. At the very least, claims about the relative importance of one form of fairness over others need to be supported by careful investigation and reporting of the measurement properties of the sub-components of fairness and the statistical considerations that these raise.

### Relational models of legitimacy

Controversy about the procedural/distributive distinction aside, it is clear that police fairness matters a great deal to people and affects the extent to which they are willing to defer to and cooperate with them. According to the group engagement model (GEM) [7], the reason the fairness of authorities affects one’s relationship with them is that fair versus unfair conduct conveys information about one’s standing in the group. Being treated respectfully by authorities and having a say in their decisions makes us feel that we are valued members of the relevant social group. Conversely, being treated unfairly undermines both our sense that the group respects us and our pride in belonging to a valued group, and thus leads to dis-identification and a loss of commitment. As such, the GEM proposes group identification as the key psychological mechanism mediating the relationship between fair treatment and people’s sense of obligation to cooperate and defer to group authorities. The model is supported primarily by evidence on employee-management relations in organisational contexts [13,28]. However, there is also supportive evidence from contexts involving the courts and the police, in which the relevant group membership is understood to be the nation or society as a whole. One early investigation found pride and respect pertaining to nationality to mediate the relationship between the fairness of the U.S. Supreme Court and commitment to the nation [29]. More recent studies suggest that identification as a citizen or member of society mediates the relationship between experiences of policing and police legitimacy [17,30]. The observation that the quality of one’s encounters with authority conveys information about one’s belonging in the group is developed further in the notion of ‘misrecognition’ [31,32]. For example, accounts of British Muslims’ experiences of airport security indicate that the behaviour of security officers can serve to position them as outsiders, often in violation of their own sense of Britishness [32]. In short, then, experiences of unfairness with the police can be expected to undermine identification with wider society, which in turn would undermine legitimacy and thus cooperation and compliance with the law.

### The relationship between authorities and groups

We suggest, however, that identification is unlikely to be the whole story as to why fairness affects police legitimacy, for two reasons. First, even if the GEM explains why being treated unfairly oneself would lead to dis-identification, it does not explain the effect of others being treated unfairly. That is, seeing the police as unfair in general, independently of whether or not one is on the receiving end of this unfairness, also undermines legitimacy, yet cannot be due to inferences about one’s own standing in the group [6,12,14,16,30,33]. Second, in the small number of studies that test the identification mediation hypothesis in the context of policing, the effect of identification on legitimacy is small in comparison with the direct effect of fairness [17,30]. This implies that fairness also leads to legitimacy primarily via other mechanisms. Third, while unfairness may lead to a psychological distancing of oneself from the group (in this case, dis-identification as a citizen or member of society), it is equally possible that one would exclude the authority as not being genuinely of the group, whilst maintaining one’s own claim on group membership. In other words, one can exclude the unfair authority rather than excluding oneself. This possibility may be less apparent in organisational settings because of the close connection between how one feels treated by one’s manager or supervisor and how one feels treated by the organisation that one works for: to a certain extent they could subjectively represent the same source of unfairness. However, in the context of police-citizen encounters, we suggest that unfair conduct by police officers could say more about the ingroup versus outgroup status of the police force than about one’s own belonging.

The social identity theory of leadership [34] proposes that leadership is a function of group processes whereby an effective leader is one that is seem to embody group norms and interests. Haslam et al. argue that to the extent that leaders ‘do it for the group’–that is, that their agenda is inseparable from the realisation of group goals, engaging as a follower is a way of enacting one’s social identity. We suggest that the relationship between legitimate institutions and groups is akin to that between leaders and groups, in that their legitimacy depends on their ability to embody group norms and work for group interests. Scholars revisiting Milgram’s classic obedience studies have noted that obedience to authority in that paradigm can be understood in terms of the ‘experimenter’ creating a psychological group that includes himself and the ‘teacher’ engaged in a shared scientific agenda [35]. Similarly, in the context of the social psychological dynamics of protest and of social change, it has been argued that minorities in conflict with authorities such as governments can only gain the solidarity of majority groups to the extent that members of the latter view themselves as part of a common category with the minority, and the authority as outgroup [36]. Thus, the workings of particular institutions can be seen as aligned or not with one’s group, with consequences for its ability to wield legitimate authority.

A number of existing lines of research on police legitimacy support this view and highlight the importance of variability in the relationship between the authority and the group. For example, a so-called neo-Durkheimian perspective on police legitimacy argues that people evaluate the police on the extent to which they act in the group’s best interests by representing and defending the moral order of the community [11,18,37]. Similarly, Jackson and Bradford [38] advance a notion of trust in the police that entails a perception of them as sharing the same agenda as the community as a whole. Such alignment between police and public agendas–termed variously as ‘moral solidarity’ [12], ‘trust in community engagement’ [8] or even ‘social identification’ [18]–all capture the variability in the extent to which the police are viewed as representatives of group priorities and agency. The construct has been found to powerfully predict police legitimacy in United States and United Kingdom contexts [12,18,39], although it is measured in a way that seems more indicative of value similarity between individual survey respondents and the police [12,18].

Moreover, to the extent that crime is experienced as a breakdown of social cohesion and the failure of the police to uphold the moral order, it may matter little from a theoretical point of view whether these goals are ‘instrumental’ (e.g. crime reduction) or ‘symbolic’ (e.g. representing group morals), since these concerns are bound together in practice and the relative weighting of each depends on the particular context [18,40]. What does matter is that the authority, in this case the police, is seen as being *of* the group, being on the ‘same side’, rather than external to it or aligned with ulterior outgroup interests. It is this alignment in goals and priorities between the group and the authority that is theoretically crucial, and we shall therefore refer to this relationship as ‘goal alignment’ between the police and wider society. Thus, independent of any relation to an individual’s identification with the group, experiences with the police can be expected to affect legitimacy by determining the extent to which they are seen as aligned to group interests.

### Bounded authority

While insights into leadership can be usefully applied to understanding legitimate authority, there is also an important distinction to be drawn between leadership and authority. Leaders are persons, and thus are held to personify the group–i.e. to be personally prototypical of the group and so forth. By contrast, authority is characterised by its bureaucratic quality, operating through impersonal institutions. The legitimate power of authorities is wielded by people who have been designated to particular roles that empower them in specific ways. Thus, the basis of these incumbents’ power it is not their ability to personify the group as prototypical members, but rather their designation through normatively accepted procedures. Thus, it is the system rather than any particular individual that must be seen to realise group goals. While these people may be referred to conventionally as ‘leaders’ in a general sense, their influence need not necessarily be of the sort described by psychological theories of leadership. In Weber’s [41] terms, this is akin to the distinction between ‘charismatic’ authority, in which power resides in a person, and ‘legal’ authority, in which it operates through systems and practices. Thus, police officers are designated to a role and their power is contingent on that designation according to accepted norms, conventions and laws.

The fact that incumbents of authority must be designated by normatively accepted practices generally imposes limits on what they can do: it limits at the same time that it empowers. This quality of simultaneous empowerment and constraint has been termed ‘bounded authority’ [33]. A corollary of bounded authority is that incumbents’ conduct can be assessed against the expectations that accompany their designation, and, where it deviates from these, legitimate power is undermined. Thus, the ability of police officers and so forth to wield authority is contingent on the ‘belief that [they] act according to societal expectations of rightful conduct in their use of authority’ [33]. Independent of the relation between oneself and group, or group and institution, incumbents may therefore lose legitimate power if they are generally seen to be outside the accepted standards that bind their authority. This suggests a third route from experience to illegitimacy: the perceived fairness of police officers in general may be undermined by experiences of conduct beyond acceptable boundaries of their power, and will directly undermine legitimacy.

### Young people and the police in Northern Ireland

In the present study we test the relationships between experiences of policing on the one hand and legitimacy, cooperation and compliance with the law on the other. Specifically, we examine three potential pathways leading from experience to legitimacy and thus to cooperation and compliance. The first is the pathway proposed by GEM, whereby the experience of unfair conduct undermines identification with the group that the authority is supposed to represent (in this case, wider society), leading to a loss of legitimacy. Second, beyond GEM, we propose that such experiences undermine goal alignment such that the police are seen to serve interests other than those of the ingroup. Third, we propose that experiences of unfairness in direct encounters with the police lead to a broader evaluation that the police in general treat people unfairly, and that this in turn directly undermines legitimacy.

We test these expected pathways in the context of police-youth relations in Northern Ireland. The issue of police legitimacy is especially pertinent in Northern Ireland because of the history of violent conflict there, particularly during the period known as the ‘Troubles’ between the late 1960s and 1990s. Grievances against the police, linked to an alienation from the state among nationalists and Catholics, were central to the armed Irish Republican campaign. Police officers were frequent targets of Republican violence and were protagonists in the conflict, and policing reform has been a key element in the peace process [42]. This has included addressing the substantial underrepresentation of Catholics among police officer that characterised the police force until then. However, despite this background, surveys since the ceasefires and the Good Friday Agreement indicate fairly widespread dissatisfaction as well as negative contact with the police among young people, irrespective of their particular ethno-sectarian (i.e. Protestant or Catholic) community background [43–45]. Given the political context, we therefore need to attend to the potential demographic determinants of perceptions of the police among these young people. Nonetheless, our primary concern is with processes and dynamics that would be equally applicable in other contexts with or without recent histories of political violence.

## Method

### Participants

Eight-hundred-and-thirty 14- to 16-year-olds were recruited to participate in the study by completing the questionnaire under teachers’ supervision in their school classrooms. Of these 819 provided sufficiently complete responses to be included in the dataset analysed here. All secondary schools of all types in Northern Ireland were invited by post to participate in the study, and all thirteen that responded positively were included. A slight majority of participants (57.6 percent) were female. The numbers of Catholic and Protestant participants were similar (38.7 percent and 42.5 percent respectively). The proportion of participants indicating entitlement to free school meals (indicating very low family income) was similar to that found in the general population of that age (15.1 percent compared to 15.8 percent). Thus, our sample was broadly representative of the population from which it was drawn in terms of key socially relevant demographic characteristics.

### Ethical approval and informed consent

The study was approved by the School of Psychology Research Ethics Committee at Queen’s University Belfast, which reviewed all of the materials and procedure. After securing the agreement of schools to collect data, parents of all potential participants were sent a letter that explained the nature and purpose of the study. In most schools, we used an opt-in system of parental consent whereby parents needed to return a signed consent form positively granting consent in order for their child to participate. In a small number of schools, head teachers advised that an opt-out system was more appropriate, and we therefore took this approach after gaining specific approval to do so from the ethics committee. In addition, participants themselves were provided with a brief written explanation of the study and what it would involve, including that they could withdraw at any time if they wished. Participants then explicitly indicated their agreement to participate by checking a box to this effect.

### Measures

A complete list of questionnaire measures is provided in S1 Appendix.

#### Experiences with the police

Participants were asked whether they had ever spoken to, or been spoken to by, a police officer. Those answering affirmatively were then asked to complete a set of items measuring aspects of this encounter, thinking of the occasion that they remembered best. These items were drawn from those used in earlier survey research on young people in Northern Ireland [43]. Participants responded to the experience items on a 5-point scale where 1 indicated ‘not at all’ and 5 indicated ‘very much so’. The items covered both police conduct and participants’ own emotional experiences during the encounters.

Participants indicated the extent to which the police officer(s) involved in the encounter were ‘respectful’, ‘helpful’, ‘professional’, ‘considerate’, ‘fair’, ‘polite’, ‘rude’, ‘unfair’ and ‘insulting’. They also indicated the extent to which the police officer wrongly accused the participant, assumed they were ‘up to no good’, picked on them and swore at them. Participants also indicated the extent to which they felt ‘scared’, ‘humiliated’, ‘angry’, ‘threatened’, ‘embarrassed’, ‘listened to’, protected’, ‘proud’, ‘respected’, ‘understood’, ‘confident’ and ‘safe’. These measures of experiences with the police were subsequently used to derive five subscales of experiences based on exploratory factor analysis described below.

#### Identification with wider society

Identification was measured using the investment scale of Leach et al.’s [46] comprehensive measure of social identification, which was reworded such that the referent group was ‘this society’ or ‘wider society’. The investment measure includes three subscales: solidarity (α = .88; e.g. ‘I feel a bond with wider society’), satisfaction (α = .88; e.g. ‘Being a member of this society gives me a good feeling’) and centrality (α = .84; e.g. ‘The fact that I am a member of this society gives me a good feeling’). The overall identification scale was reliable (α = .91).

#### Police fairness

Measures of police fairness were adapted from Reisig, Bratton and Geertz’s (2007), who compiled them from items typically used in published studies of the police procedural justice and legitimacy by Tyler and colleagues [1,11,12,16]. Minor edits were made to these items through consultation with small groups of young people in order to make them as easily comprehensible as possible to people from the target age group with a varied levels of educational attainment. For example, ‘The police make sure citizens receive the outcomes they deserve under the law’ was reworded as ‘The police make sure people get what they deserve under the law’.

The fairness items tapped three aspects of police fairness as a general perception, rather than judgements of a specific encounter: respect (α = .91; 5 items, e.g. ‘The police treat people with respect), fair decision-making (α = .74, 5 items; e.g. ‘The police make their decisions based on the facts of the situation’) and distributive fairness (α = .79; 5 items, e.g. ‘The police enforce the law equally with all people’). The overall scale for fairness was also reliable (α = .92).

#### Police goal alignment with wider society

Three items were generated for the present study to measure the extent to which the police are seen to serve to benefit wider society [‘In general, when the police succeed in their objectives, wider society benefits’, ‘What the police do generally benefits wider society’, ‘Whomever the police are meant to serve, they definitely do not serve wider society’ (negatively worded)].

#### Legitimacy

Four items were drawn from Reisig et al. [23] to measure legitimacy, defined specifically as an obligation to obey the police (α = .73; e.g. ‘You should accept police decisions even if you think they are wrong’).

#### Cooperation with the police

Eight items were drawn from Reisig et al. [23] to measure the extent to which participants would be willing to actively cooperate with the police, such as by reporting crime, accidents and so forth. For each item, participants indicated the extent to which it was ‘the kind of thing I would do’ on a 5-point scale (1 = ‘not at all the kind of thing I would do’; 5 = ‘very much the kind of thing I would do’, α = .77).

#### Compliance

The items measuring compliance represented a combination of items taken from Reisig et al. [23] and Hamilton et al. [43], tapping participants’ self-reported likelihood to engage in a variety of minor crimes and anti-social behaviours that are relevant to our particular sample. Responses were given on the same 5-point scale as the cooperation items (α = .85).

#### Demographic control variables

Participants reported their sex, age, community background (Protestant or Catholic) and entitlement to free school meals, which we used as control variables in the main analysis.

## Results

### Descriptive statistics

Mean, standard deviations and correlations for all manifest variables are reported in Table 1.

**Table 1:** Descriptive statistics and correlations for main variables of interest.

	Mean (S.D.)	1.	2.	3.	4.	5.	6.	7.	8.	9.	10.	11.
1. Fair conduct	3.79 (1.02)	-	.75	-.49	-.62	-.44	.19	.65	.53	.49	.32	-.38
2. Positive affect	1.57 (.84)		-	-.46	-.54	-.37	.27	.65	.51	.48	.38	-.31
3. Negative affect	3.37 (1.06)			-	.54	.47	-.14	-.41	-.33	-.27	-.23	.29
4. Suspicion	1.89 (1.19)				-	.49	-.22	-.51	-.40	-.42	-.27	.29
5. Hostility	1.23 (.67)					-	-.18	-.41	-.33	-.38	-.25	.35
6. Identification with wider society	2.72 (.80)						-	.30	.23	.24	.18	-.14
7. Police fairness	3.39 (.77)						.31	-	.67	.62	.41	-.37
8. Police goal alignment	3.35 (.90)						.22	.66	-	.59	.31	-.37
9. Police legitimacy	3.44 (.74)						.23	.61	.58	-	.42	-.42
10. Cooperation with the police	3.44 (.94)						.21	.39	.30	.38	-	-.23
11. Noncompliance with the law	1.60 (.69)						-.12	-.34	-.34	-.39	-.20	-

Correlations shown above the diagonal are for 664 participants reporting a direct encounter with the police. Those below the diagonal are for the full sample.

### Dimensions and types of experiences with the police

The measures of direct experience with the police were adapted from earlier descriptive research without specific expectations regarding their dimensionality. In order to explore this and derive scales, we entered all of the items into exploratory factor analysis with principal axis functioning and Oblimin rotation (S1 SPSS syntax). Five factors were extracted with eigenvalues of above 1, with the eigenvalue dropping to .87 for the sixth factor. We therefore extracted five factors. Inspection of the Oblimin rotated solution (Table 2) indicates the following five dimensions of experience with police officers, on the basis of which we computed five subscales: fair conduct (α = .90), negative affect (α = .81), positive affect (α = .91), suspicion (α = .83), and abuse (α = .66). Items not loading at least .50 on any factor, and items with cross-loadings great than .30 were excluded from the computation of the scales.

**Table 2:** Rotated (Oblimin) factor loadings of experience items on five factors extracted with principal axis functioning.

	Factor				
	1	2	3	4	5
**Fair conduct**					
Considerate	**-.710**				
Respectful	**-.685**				
Fair	**-.676**				
Unfair ^a^	**.649**			-.308	
Polite	**-.622**				
Insulting ^a^	**.566**				-.404
Professional ^a^	**-.563**		.308		
Rude	**.554**				
Helpful ^a^	**-.504**		.376		
Slow to respond ^a^	.445				
Aggressive ^a^	.441				-.370
**Negative feelings**					
Humiliated		**.816**			
Embarrassed		**.806**			
Scared		**.792**			
Threatened		**.616**			
Angry ^a^		.422		-.329	
**Positive feelings**					
Confident			**.744**		
Proud			**.743**		
Safe			**.730**		
Protected			**.670**		
Understood			**.654**		
Respected			**.651**		
Listened to			**.594**		
**Suspicion**					
Followed their own rules and procedures properly ^a^	-.310		.362		
Assumed you were up to no good				**-.770**	
Wrongly accused you				**-.725**	
Picked on you for no reason				**-.656**	
Deliberately Provoked you ^a^				-.485	-.444
Harassed ^a^				-.302	
**Abuse**					
Used discriminatory language					**-.855**
Swore at you					**-.696**
Abused their Power ^a^				-.369	-.418

Values < .30 are suppressed.

^a^ Items not used to compute experience scales due to cross loadings and/or not loading > .50 on any factor.

Latent class analysis (LCA) enabled us to examine the specific patterns of co-occurrence of the five dimensions of experience in the form of discrete types of encounter with the police. As well as participants reporting no encounter with the police, three participants with missing data on one or more of the five dimensions were excluded from this part of the analysis, leaving a total of 664. We conducted the analysis in an exploratory fashion by first specifying a series of two-, three-, and four-class models each with the subscales for the five dimensions as indicators (S1–S3 MPlus command). The two-class model showed high classification accuracy, indicated by high class probabilities for the assigned classes (.96 to .99) and low probabilities for non-assigned classes (.01 to .04). By comparison, the three-class model showed significantly better relative fit (LR difference = 508.78, p < .001) and similar classification accuracy with high probabilities for assigned classes (.93 to .98) and low probabilities for non-assigned classes (.00 to .03). The four-class model showed a further improvement in fit compared to the three-class model as would be expected with the addition of a latent class (LR difference = 289.65, p < .001) but with a slight reduction in classification accuracy with lower probabilities for assigned classes (.88 to .96) and some higher probabilities for non-assigned classes (.00 to .09). We thus selected between the three- and four-class models on the basis of interpretability [47]. Inspection of the estimated mean values of the five indicators for each class indicated that the addition of a fourth class was not readily interpretable because it was simply an intermediate position between two existing classes rather than a distinctive pattern. Accordingly, we proceeded with the three-class model.

**Fig 1:**
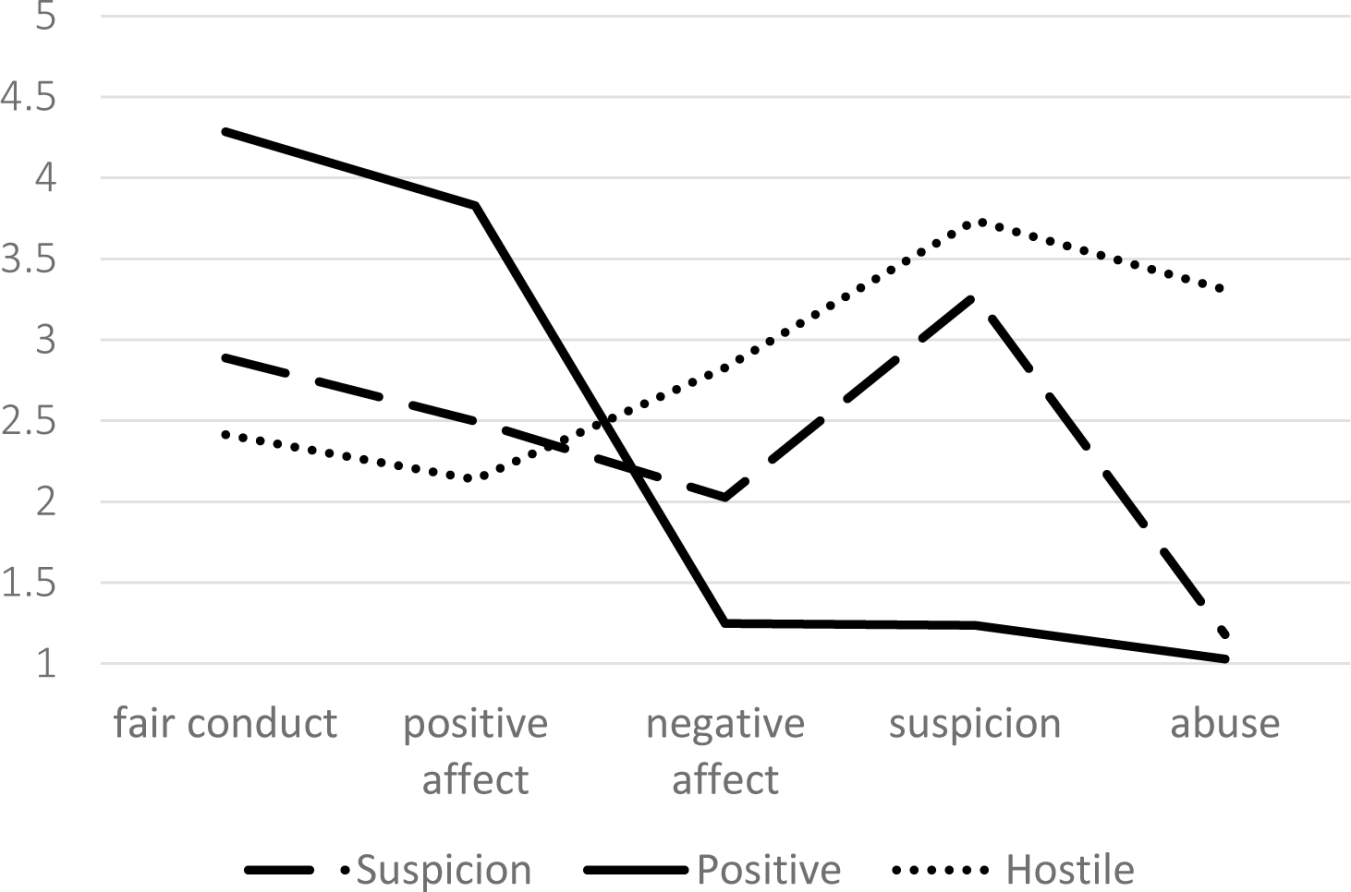
Estimated values for each of the dimensions of experience derived from three latent classes.

Estimated values derived from the three-class model are illustrated in Fig 1. Class 1 includes 455 participants and is characterised by high values on the positive dimensions (fair conduct, positive affect) and low values on the negative dimensions (negative affect, suspicion and abuse). Class 2, which includes 55 participants, showed a reverse pattern with low values on the positive dimensions and high values on the negative dimensions. Class 3 includes the remaining 154 participants and moderate values for fair conduct, positive affect and negative affect, but with values more similar to class 2 for suspicion and more similar to class 1 for abuse. Class 1 is thus labelled as ‘positive’, class 2 as ‘hostile’ and class 3 as ‘suspicion’ on the grounds that being subjected to (perceived) unwarranted suspicion is the distinctive negative dimension for this class.

### Factor structure of police fairness

**Fig 2:**
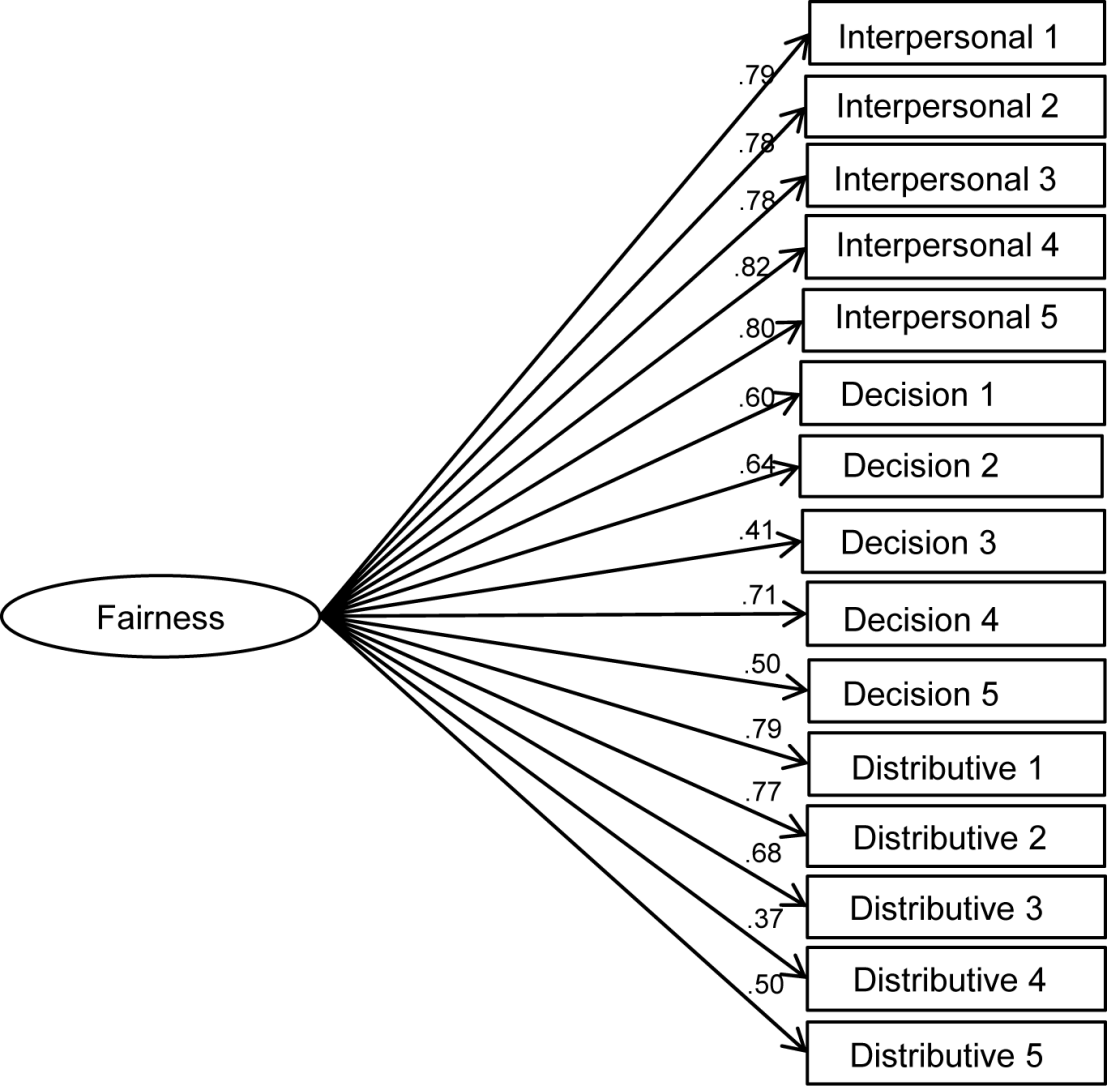
One-factor model of police fairness measures (general fairness; model A).

**Fig 3:**
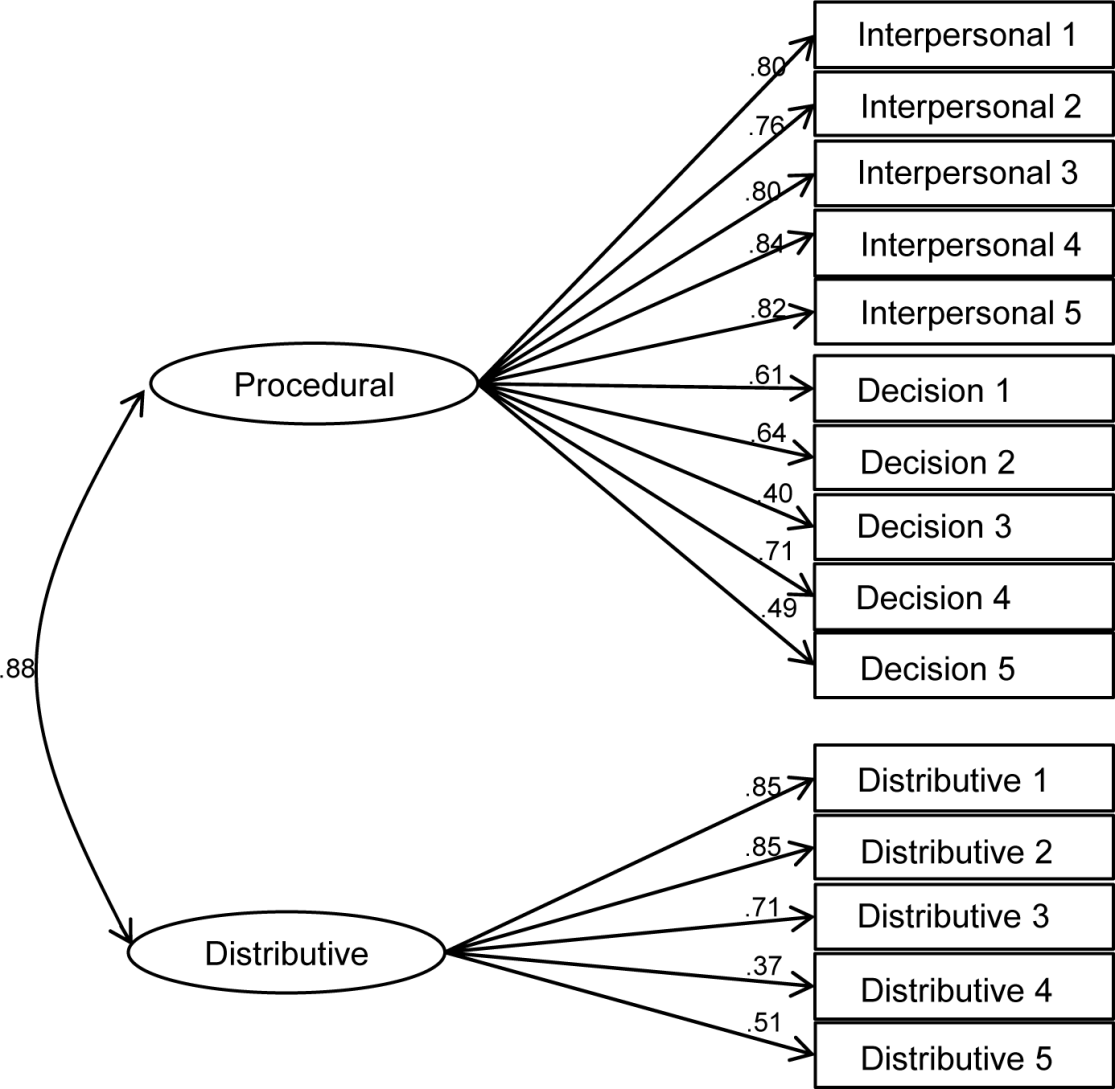
Standard two-factor model of police fairness (procedural and distributive fairness; model B).

**Fig 4:**
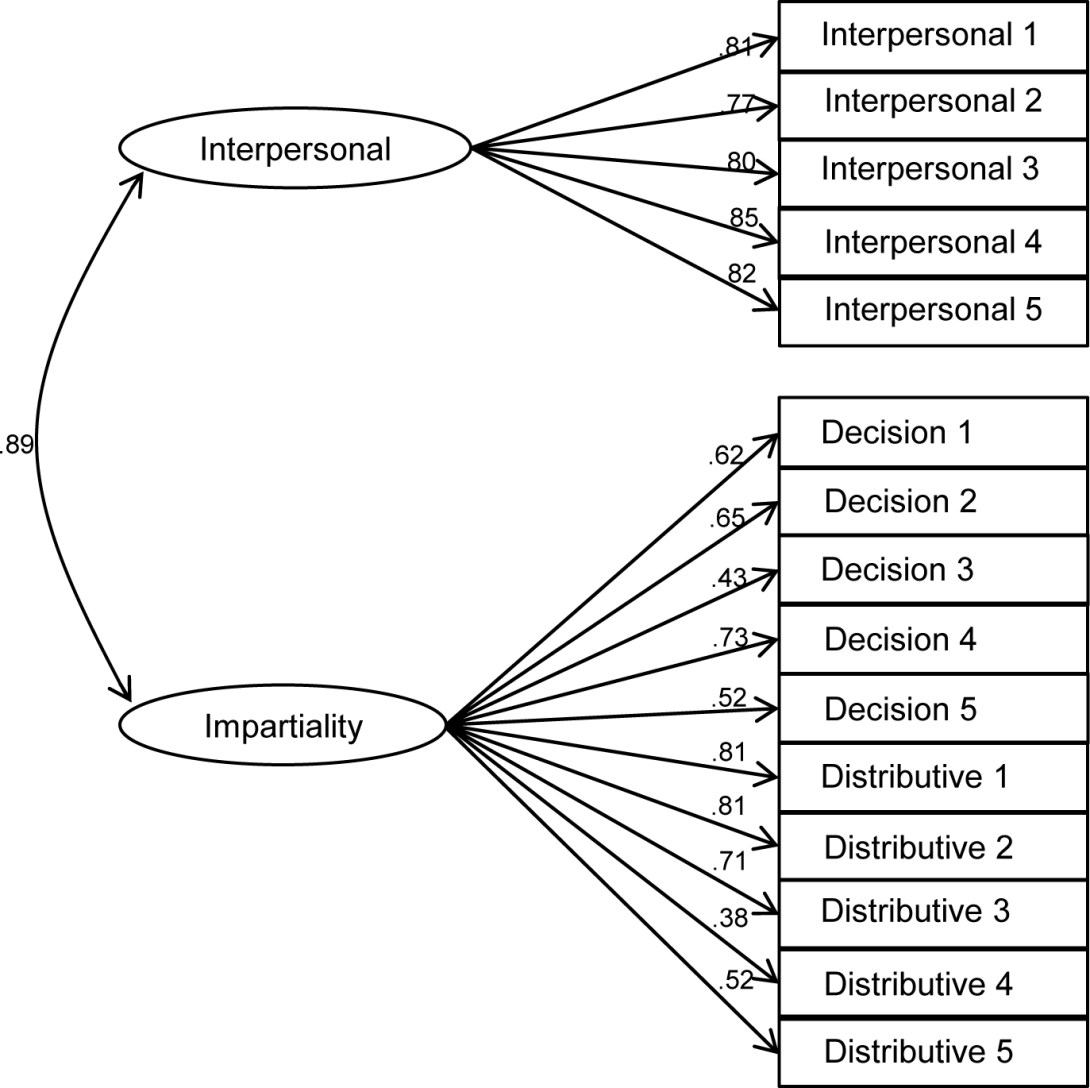
Alternative two-factor model of police fairness (interpersonal respect and impartiality; model C).

**Fig 5:**
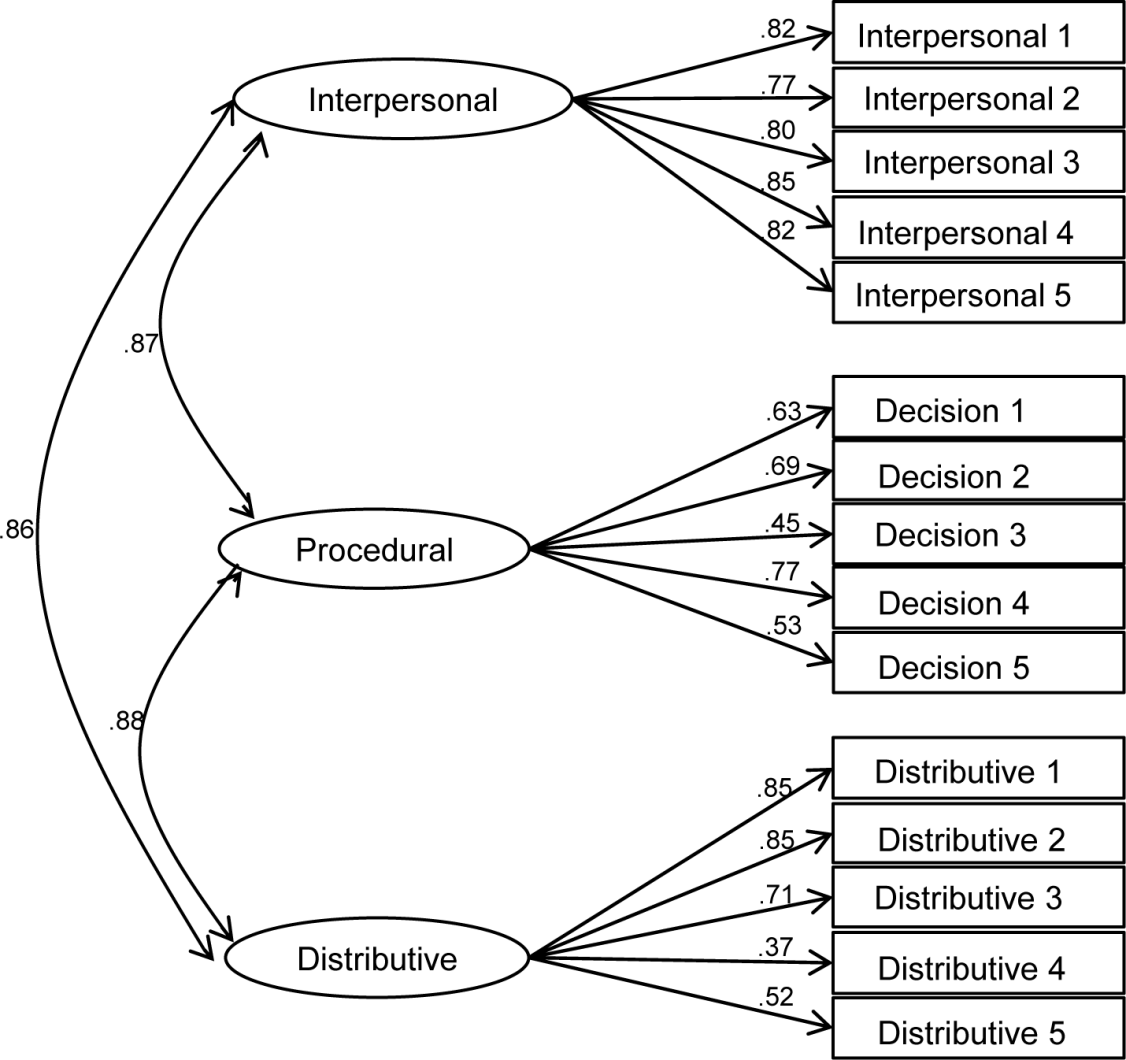
Three-factor model of police fairness (interpersonal respect, fair decisions and distributive fairness; model D).

Given the challenges identified in the literature to the standard measurement structure of procedural and distributive fairness [23,24,26], and in order to ascertain the most appropriate conceptualisation of these constructs for our main analysis, we tested the relative fit of four competing measurement models that might plausibly account for the covariance among the 15 police fairness items using confirmatory factor analysis in MPlus 7. Model A specifies all items as indicators of one general ‘fairness’ factor (Fig 2). Model B (Fig 3) is the standard model suggested by Tyler and colleagues’ conceptualisation [1,15,16] with two factors: procedural fairness, which includes interpersonal respect and fair decision making items, and distributive fairness. Model C is an alternative two-factor model that assumes decision making and distributive fairness items to be indicators of one ‘impartiality’ factor, with interpersonal respect items loading separately (Fig 4). Finally, model D specifies three separate factors for interpersonal respect, fair decision making and distributive fairness (Fig 5).

**Fig 6:**
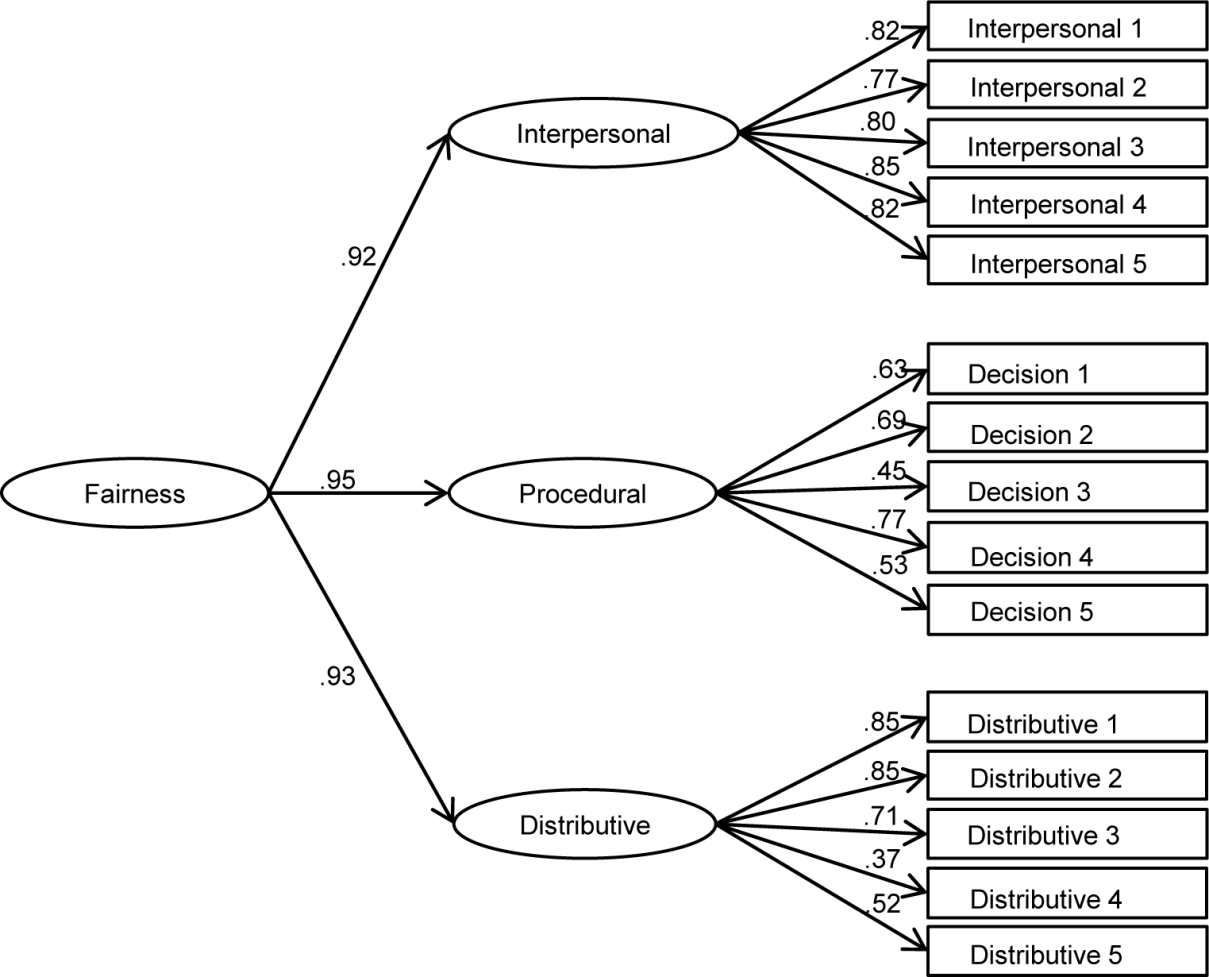
Higher-order factor model in which interpersonal respect, fair decisions and distributive fairness indicate a superordinate fairness factor (model E).

Fit indices for each of the measurement models are presented in Table 3. Analysis of chi-square differences indicates that both 2-factor models, B (Δ χ^2^ (1) = 148.58, p < .001) and C (Δ χ^2^ (1) = 202.80, p < .001), show significantly better fit than model A, the 1-factor model. Models B and C cannot be compared using the chi-square difference because they are not nested. Nonetheless, examination of BIC statistics suggests that model C shows superior fit to model B. In turn, the chi-square difference between models D and C (which are nested) suggests that model D has better fit (Δ χ^2^ (2) = 68.05, p < .001). Given the very high correlations between the three factors (all exceeding .85), it is appropriate to specify the three factors in model D as subordinate to a general fairness factor in a higher-order factor model (model E). The resulting hierarchical model is illustrated I Fig 6 and its fit indices are identical to those of model D.

**Table 3:** Fit indices and comparisons between the four measurement models for police fairness.

	Model A	Model B	Model C	Models D & E
RMSEA	.091	.080	.075	.069
CFI	.905	.928	.937	.947
TLI	.889	.915	.925	.936
BIC	31378.62	31236.74	31182.52	31127.89
χ^2^ (d.f.)	699.53 (90)	550.95 (89)	496.73 (89)	428.68 (87)

### Mediated paths from experience to cooperation and compliance

In order to test the hypothesised mediated paths from experiences with the police to cooperation and compliance we estimated a path model with latent factors in which identification, fairness and police goal alignment were specified as parallel mediators of the paths from experience to legitimacy, which in turn was specified as a predictor of cooperation and compliance. The model was estimated using MPlus 7 (S4 MPlus command).

Experiences with the police were modelled using the latent classes obtained in the LCA above. These were dummy coded such that participants who did not report any interaction with police officers served as a baseline group against which those in each of the three latent classes were compared.

On the basis of the CFA reported above, respect, fairness and distributive fairness were specified as indicators of a general fairness factor. Solidarity, satisfaction and centrality were indicators of the identification factor. Police goal alignment was indicated by the three single items in that scale. Items measuring legitimacy, cooperation and compliance were used to compute items parcels that were the indicators of those respective factors.

We controlled for gender and community background (dummy coded with Protestant background is the baseline, compared to Catholic background and ‘neither’) by including them as predictors of all of the endogenous variables. These paths are omitted from the diagram for clarity of presentation. Family income (indicated by entitlement or not to free meals) was not included as a control variable in the model reported here because there were 36 cases with missing data on this variable, which would have reduced the usable sample size. However, an alternative model that does include free meals as a control variable produces almost identical estimates (available on request). Thirteen respondents had missing data for gender and/or community background, leading to a sample size of 806 for the SEM analysis.

**Fig 7:**
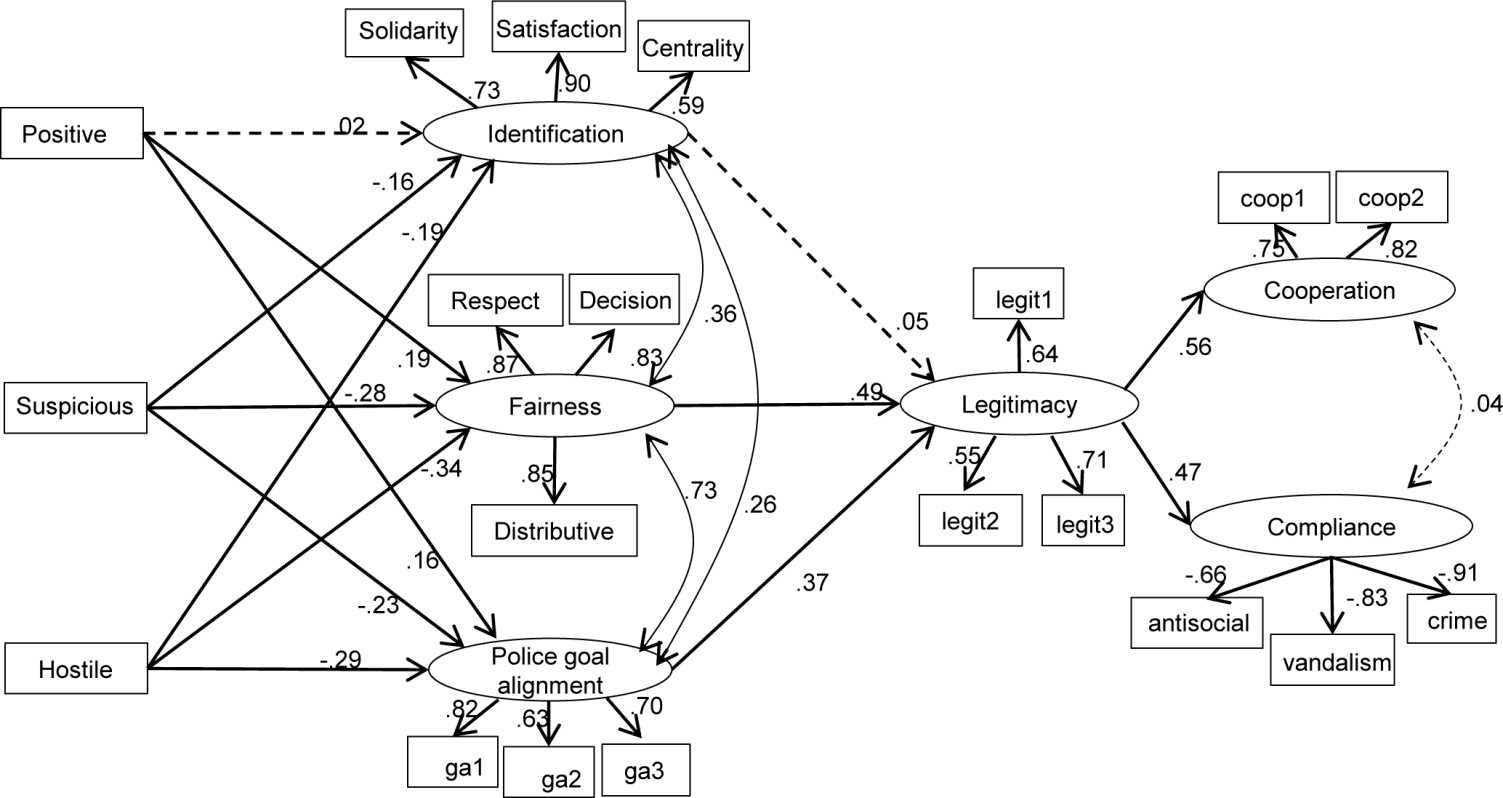
Structural equation model specifying mediated paths from experience to cooperation and compliance.

The model with estimated path coefficients is shown in Fig 7. The model shows good fit (RMSEA = .033, CFI = .974, TLI = .966, SRMR = .031). It indicates negative paths from both types of negative encounter (suspicious and hostile) to identification, fairness and police goal alignment. Positive encounters predict perceived fairness and goal alignment but not identification. The model also shows independent paths between identification, fairness and goal alignment on the one hand and legitimacy on the other. Finally, legitimacy positively predicts cooperation and negatively predicts noncompliance.

Female participants reported higher levels of legitimacy (B = .19, 95% CI = .08/.30) and high compliance (B = .19, 95% CI = .08/.30) than male participants. Other than this there were no statistically significant paths between the demographic and endogenous variables in the model.

#### Mediation analysis

Table 4 shows the estimated indirect paths from experiences to cooperation and compliance with standard errors based on 1000 bootstrap samples. In the case of both cooperation and compliance, these indicate significant paths from positive experiences via fairness and legitimacy. There were also significant paths from both positive and suspicious experiences via fairness and legitimacy, and via goal alignment and legitimacy. No indirect paths involving identification were significant.

**Table 4:** Indirect paths from three types of experience with the police to cooperation and noncompliance.

	Cooperation		Compliance	
	Beta	95% C.I.	Beta	95% C.I.
**Positive Experience**				
Via identification-legitimacy	.001	(-.007, .009)	.001	(-.093, .009)
Via fairness-legitimacy	**.082**	**(.016, .149)**	**.051**	**(-.093, -.009)**
Via goal alignment-legitimacy	.055	(-.005, .115)	-.034	(-.072, .003)
**Negative experience**				
Via identification-legitimacy	-.018	(-.059, .023)	-.011	(-.036, .014)
Via fairness-legitimacy	**-.300**	**(-.516, -.084)**	**-.186**	**(-.322, -.051)**
Via goal alignment-legitimacy	**-.194**	**(-.359, -.029)**	**-.120**	**(-.225, -.015)**
**Suspicious experience**				
Via identification-legitimacy	-.010	(-.033, .013)	-.006	(-.170, -.028)
Via fairness-legitimacy	**-.159**	**(-.274, -.044)**	**-.099**	**(-.170, -.028)**
Via goal alignment-legitimacy	**-.101**	**(-.194, -.008)**	**-.062**	**(-.120, -.005)**

Confidence intervals based on 1000 bootstrap samples. Bold type indicates estimates with confidence intervals not overlapping with zero.

## Discussion

In a large sample of young people in Northern Ireland, we investigated the relationship between direct experiences of policing on the one hand, and police legitimacy leading to cooperation and compliance on the other. Our results suggest two mechanisms linking experience to legitimacy. First, the perception that the goals of the police are aligned to those of wider society mediates the link between the quality of experiences and a felt obligation to obey the police. Compared to individuals who had not directly interacted with a police officer, those with experiences of hostile or suspicious encounters tended to not to see the police as acting to serve wider society, while the reverse was true for those with positive experiences. This differs from the established view in the field that experiences with authorities matter because they can support or undermine one’s own sense of belonging to the relevant group [7]. Although identification with wider society was related to experience, it did not relate independently to legitimacy. The findings support our contention that a negative encounter with authority may lead to that authority rather than oneself being placed outside the psychological group. Second, direct experiences are linked to perceptions of the police in general being either fair or unfair, which directly relates to legitimacy independently of either goal alignment or identification. This is in line with the notion of ‘bounded authority’ [33], whereby the norms underpinning the empowerment of police officers with certain entitlements (including the right to be obeyed) simultaneously constrains their conduct. If one’s experience suggests that officers step outside the accepted bounds of their authority, such as by verbally abusing people or bothering them without a good reason, then the basis for their legitimacy is undermined.

Our findings are in line with existing evidence that there must be a shared agenda between the police and the public in order for the former to be seen as legitimate by the latter [12,18,37,38]. However, to our knowledge the present study is unique in both conceptualising this alignment at the group level in terms of alignment with wider society and measuring it accordingly. Previous survey work has instead asked participants about the interpersonal value similarity between participant and police officers, which fails to capture the collective level processes implied by the theoretical accounts [12]. Moreover, we have tested goal alignment as a direct alternative to the identification-mediated pathway that prevails in the social psychological literature and found evidence for the former and not the latter. To date, most evidence for group identification mediating the link between fair treatment and legitimacy has come from research on organisation-employee relations rather than police-public relations, although there are some recent exceptions to this [17,30]. The goal alignment and identification pathways are not necessarily antagonistic and each may well be more relevant in any given context. It must of course be noted that analyses of cross-sectional correlational data cannot confirm the direction of the causal pathways, and that this would require experimental of longitudinal evidence, and possibly alternative statistical methods of mediation analysis. Nonetheless, our contribution here is to highlight that there is more than one plausible way in which experiences of authority can affect how people represent the relationships between themselves, the police and society, and thus more than one way in which social identity processes can support or undermine police legitimacy. This in turn should lead to an enriched theoretical account of the multiple conditions needed for legitimate authority to function, and hence the multiple ways in which legitimacy can be lost: (1) an individual may dis-identify with the relevant group; (2) the authority may be seen as serving interests other than the ingroup that it is supposed to serve; (3) incumbents such as police officers may act outside the bounds of the authority and to the extent that this is seen as a systematic problem the legitimacy of the wider institution is undermined. Thus, we suggest that our findings point the way for a thorough investigation of all of these routes to illegitimacy.

A secondary contribution of the current study is to help to clarify the nature of the relationship between different kinds of fairness that have been identified and contested in the literature [23]. While procedural and distributive fairness have often been measured and pitted against each other using survey data, the adequacy of the measurement structure implied in such studies has rarely been tested directly. Our confirmatory factor analysis based on typical measures of police fairness found in the literature finds little support for the usual dichotomy between procedural and distributive fairness. To the extent that a two-factor conceptualisation is appropriate, the data suggest that fair decision making and distributive fairness belong together as a common impartiality dimension, while interpersonal respect is a distinct second factor. This evidence is at odds with the usual practice of treating interpersonal respect as an aspect of procedural fairness and contrasting this with distributive justice. Moreover, when modelling the traditional conceptualisation of procedural versus distributive fairness, we find a correlation of .89 between the two. This suggests that any claims regarding the relative importance of procedural over distributive fairness based on studies that pit them against one another in multiple regression should be regarded with considerable caution. On the basis of these findings, we concur with Hauenstein et al.’s [24] advice that a general ‘fairness’ construct is more appropriate if the aim is to predict outcomes of fairness.

With regard to the measurement of direct experience with the police, we adopted a data driven approach by exploring the factors underlying an extensive set of items drawn from previous research in this context [43] and then deriving a set of three distinct types of experience using LCA. Of interest here is the dimension relating to a sense of being accused or picked on without justification. Our latent class analysis identified a group of 154 participants (23 percent of those reporting some encounter with the police, and 74 percent of those reporting a negative encounter) who scored relatively highly on this dimension whilst reporting almost no verbal abuse in contrast to the other class of participants reporting a negative encounter. In other words, our analysis indicates that for a majority of those young people in our sample reporting a negative experience with the police, the salient negative feature of their interaction was that the officer(s) treated the participant as problematic (e.g. criminal or ‘up to no good’) in a way that felt unjustified. This is significant particularly if we consider recent work on misrecognition in encounters with authority [31,32]. Here, the problem seems to be not so much a specific unfair decision or lack of civility in the interaction but rather being positioned by the authority in a way that is at odds with one’s own understanding of oneself. To consider oneself as decent, law abiding member of society yet be treated as a problem by the police therefore appears to be a distinct and consequential experience in the present study, and one that merits further attention.

## Supporting information

S1 Appendix Complete scales for police fairness, goal alignment, identification with society, cooperation with police and legitimacy. (PDF)

S1 Raw Data Complete raw data file included all variables used in the analyses. (SAV)

S1 SPSS syntax SPSS syntax file for scales, items parcels and factor analysis. (SPS)

S1 MPlus command MPlus command file for the 2-cluster LCA. (INP)

S2 MPlus command MPlus command file for the 3-cluster LCA. (INP)

S3 MPlus command MPlus command file for the 4-cluster LCA. (INP)

S4 MPlus command MPlus command file for full SEM model. (INP)
