# Supplementary material for: Beyond group engagement: Multiple pathways from encounters with the police to cooperation and compliance in Northern Ireland
Source: PLoS One. 2017 Sep 7;12(9):e0184436. doi: 10.1371/journal.pone.0184436 (PMC5589227; doi:10.1371/journal.pone.0184436)
Supplement: S1 Appendix — (PDF) [file pone.0184436.s001.pdf]

Complete scales for police fairness, goal alignment, identification with society, cooperation with police and legitimacy

**Police fairness**	The police treat people with respect (Interpersonal 1)<br>The police take time to listen to people (Interpersonal 2)<br>The police treat people fairly (Interpersonal 3)<br>The police respect people's rights (Interpersonal 4)<br>The police are well-mannered to people they come into contact with (Interpersonal 5)<br>The police make decisions based upon the facts of the situation (Procedural 1)<br>The police explain their decisions to the people they deal with (Procedural 2)<br>The police make decisions based on their own personal opinions (Procedural 3)<br>The police make decisions to handle problems fairly (Procedural 4)<br>The police don't listen to all of the people involved before deciding what to do (Procedural 5)<br>The police provide the same quality of service to everyone (Distributive 1)<br>The police enforce the law equally with all people (Distributive 2)<br>The police make sure that all people get what they deserve under the law (Distributive 3)<br>The police provide better services to wealthier people (Distributive 4)<br>The police give people who are not White less help because of their race (Distributive 5)	1 (strongly disagree) – 5 (strongly agree)
**Goal allignment**	What the police do generally benefits wider society (ga1)<br>Whomever the police are meant to serve, they definitely do not serve wider society (ga2)<br>In general, when the police succeed in their objectives, wider society benefits (ga3)	1 (strongly disagree) – 5 (strongly agree)
**Identification with Society**	I feel a bond with wider society (idbond)<br>I feel united with wider society (idunited)<br>I feel dedicated to wider society (iddedicated)<br>I am glad to be a member of wider society (idgladmember)<br>I think that this society has a lot to be proud of (idproud)<br>It is pleasant to be a member of this society (idpleasant)<br>Being a member of this society gives me a good feeling (idgoodfeeling)<br>I often think about the fact that I am a member of this society (idthinkabout)<br>The fact that I am a member of this society is an important part of my identity (ididentity)	1 (strongly disagree) – 5 (strongly agree)

	Being a member of this society is an important part of how I see myself (idseemyself)	
**Co-operation with Police**	Call the police to report a crime (COOPreportcrime)<br>Report suspicious activity near your house (COOPreportsuspicious)<br>Call the police to report an accident (COOPreportaccident)<br>Provide information to the police to help find a suspected criminal (COOPinformation)	1 (not at all the kind of thing I would do) – 5 (Very much the type of thing I would do)
**Legitimacy**	It is reasonable for the police to sometimes tell people what to do<br>You should accept police decisions even if you think they are wrong<br>Disobeying the police is hardly ever right<br>The police never have any right to interfere in what people are doing<br>If someone disobeys the police and gets away with it, then good for them<br>You should do what the police tell you to do even if you disagree<br>It is difficult to break the law and still feel good about yourself	1 (strongly disagree) – 5 (strongly agree)
